# Exploring the Relationship Between Atherogenic Index of Plasma, Body Mass Index, and Fatty Liver in Obese Patients

**DOI:** 10.7759/cureus.87216

**Published:** 2025-07-03

**Authors:** Prabhakar K, Anitha Aswathanarayana, Amulya Reddy

**Affiliations:** 1 Department of General Medicine, Sri Devaraj Urs Academy of Higher Education and Research, Kolar, IND

**Keywords:** atherogenic index of plasma, body mass index, hepatic steatosis, non-alcoholic fatty liver disease, obesity, ultrasonography

## Abstract

Objective: This study aimed to estimate the body mass index (BMI) and the atherogenic index of plasma (AIP) in obese individuals, evaluate the grade of fatty liver using abdominal ultrasound, and assess the relationships among AIP, BMI, and fatty liver severity.

Methodology: This hospital-based cross-sectional study was conducted at R.L. Jalappa Hospital and Research Centre in Kolar to examine the association between three key metabolic parameters - AIP, BMI, and ultrasound-diagnosed fatty liver - in obese individuals. The study was carried out over three months from December 2024 to February 2025 and included 26 adults with a BMI greater than 25 kg/m² based on the Asian cut-off values. Participants underwent BMI calculation, fasting lipid profile testing, and abdominal ultrasound for fatty liver grading. Individuals with chronic liver disease, alcohol use, or conditions affecting lipid metabolism were excluded. AIP was calculated using the established logarithmic formula: log (triglycerides (TG)/high-density lipoprotein cholesterol (HDL-C)). Statistical analysis was performed using IBM SPSS Statistics v22 (IBM Corp., Armonk, USA), with a p-value less than 0.05 considered statistically significant.

Results: In this study, 18 (69.2%) participants were classified as obese (BMI ≥30 kg/m²), and the most common fatty liver grade was Grade II, observed in 11 (42.3%) patients. A total of 17 (65.4%) patients had moderate to severe fatty liver (Grades II and III). High AIP values (>0.21) were observed in 16 (61.5%) patients, with 23 (88.4%) showing elevated atherogenic risk. AIP levels significantly increased with fatty liver severity (p<0.05), and the mean BMI also rose progressively across fatty liver grades (p<0.05). These findings highlight strong associations between BMI, AIP, and fatty liver severity in obese individuals.

Conclusion: The results demonstrated a clear positive correlation between both BMI and AIP values and the severity of fatty liver disease in the obese population. These findings suggest that BMI and AIP are practical, cost-effective screening tools for the early detection of metabolic dysfunction-associated steatotic liver disease (MASLD). Their utility is particularly relevant in clinical settings with limited access to advanced diagnostic modalities. The study supports the integration of BMI and AIP into routine screening protocols to guide early intervention and mitigate the long-term health burden of non-alcoholic fatty liver disease (NAFLD).

## Introduction

Metabolic dysfunction-associated steatotic liver disease (MASLD) has become a significant public health concern worldwide, currently affecting approximately 25% of adults [1]. This condition represents a spectrum of liver pathologies, beginning with simple fat accumulation (steatosis) and potentially progressing through various stages, including inflammation (metabolic dysfunction-associated steatohepatitis (MASH), scarring (fibrosis), advanced scarring (cirrhosis), and ultimately liver cancer (hepatocellular carcinoma) [1,2]. This issue is particularly critical in developing nations like India, where the dual burden of infectious and lifestyle-related diseases persists. The rising prevalence of obesity and metabolic syndrome has positioned MASLD as the hepatic manifestation of underlying metabolic dysfunction.

Central obesity, especially excess visceral fat, plays a crucial role in the development of MASLD. Modern understanding recognizes adipose tissue as a passive fat reservoir and a dynamic endocrine organ that actively participates in metabolic regulation. It secretes various adipokines and proinflammatory cytokines that promote insulin resistance, inflammation, and altered lipid metabolism. In individuals with obesity, hypertrophied adipocytes and increased visceral fat contribute to elevated circulating free fatty acids. These are absorbed by the liver and converted into triglycerides (TG), leading to hepatic steatosis [3,4].

Dyslipidemia, defined as abnormal blood lipid levels, contributes to MASLD. It is commonly observed in individuals with obesity and metabolic syndrome and is known to exacerbate liver fat accumulation. The atherogenic index of plasma (AIP), calculated as the logarithm of TG to high-density lipoprotein cholesterol (HDL-C) ratio (TG/HDL-C), offers valuable insight into lipid-related cardiovascular risk. This index reflects the balance between atherogenic and protective lipoproteins and is strongly associated with cardiovascular disease, atherogenic lipoprotein profiles, and impaired insulin sensitivity [5-7].

Recent studies suggest that AIP is also associated with MASLD, particularly in individuals with obesity. Elevated AIP values may reflect more severe lipid metabolism dysfunction and greater hepatic fat accumulation, positioning AIP as a potential non-invasive biomarker for fatty liver disease [8]. Additionally, body mass index (BMI), a widely used anthropometric measure of obesity, remains a practical tool for clinical assessment. Several studies have demonstrated a positive correlation between BMI, AIP, and the incidence of MASLD [9]. However, limited data exist on the relationship between BMI, AIP, and ultrasound-based grading of fatty liver in the Indian population, particularly in hospital-based settings.

Understanding these associations may support early detection of MASLD, especially in individuals with obesity who are at higher risk. Given the growing burden of obesity-related liver and cardiovascular diseases, the identification of low-cost, non-invasive, and accessible markers such as AIP may enhance early diagnosis and risk stratification. If validated, AIP, in combination with BMI, could serve as a screening tool to identify individuals with obesity who are at increased risk for MASLD, thereby enabling earlier interventions and reducing the risk of long-term complications. Therefore, this study aimed to estimate BMI and AIP in obese individuals, evaluate the grade of fatty liver using abdominal ultrasound, and assess the relationships among AIP, BMI, and fatty liver severity. We hypothesized that higher AIP and BMI values are significantly associated with greater severity of fatty liver and that AIP may serve as a low-cost, non-invasive marker for early detection and risk stratification of NAFLD in obese individuals.

## Materials and methods

Study design and setting

This hospital-based observational study was conducted in the Department of Medicine at R.L. Jalappa Hospital and Research Centre, a tertiary care teaching hospital affiliated with Sri Devaraj Urs Medical College in Kolar, Karnataka. The study aimed to evaluate the relationship between AIP, BMI, and ultrasonographically graded fatty liver in obese adults.

Study period and population

The study was conducted over three months, from December 2024 to February 2025. The study population included obese individuals with a BMI greater than 25 kg/m² based on the Asian cut-off values. Participants were recruited from both outpatient and inpatient departments of General Medicine during the study period.

Eligibility criteria

Adults aged 18 years or older with a BMI greater than 25 kg/m² who provided written informed consent were included. Exclusion criteria were chronic liver diseases such as viral hepatitis or alcoholic liver disease, pregnancy, use of lipid-lowering or hepatotoxic medications, alcohol consumption above recommended clinical thresholds (more than 20 g/day for women and 30 g/day for men), and metabolic or endocrine disorders such as hypothyroidism or Cushing’s syndrome that could independently affect lipid metabolism.

Sample size calculation

Using the standard formula for sample size calculation in cross-sectional studies with a 95% confidence level (Z=1.96), an assumed prevalence of 97% based on a previous survey of Bhandary et al. [5], and a 7% margin of error, the minimum required sample size was calculated as 23. This number was increased to 26 to account for a possible 10% non-response rate.

Data collection procedure

Anthropometric measurements were obtained using calibrated instruments. Height and weight were recorded for each participant, and BMI was calculated using the standard formula: weight in kilograms divided by height in meters squared (kg/m²). Fasting venous blood samples were collected after a minimum fasting duration of 8-10 hours, and all samples were drawn between 7:30 AM and 9:00 AM to minimize the impact of diurnal variation. Serum TG and HDL-C levels were measured using enzymatic colorimetric methods. The AIP was calculated using the formula: log (TG/HDL-C), where both values were expressed in molar concentrations. In addition to lipid parameters, liver function tests, including alanine transaminase (ALT), aspartate transaminase (AST), and gamma-glutamyl transferase (GGT), were measured for a more comprehensive evaluation of hepatic status and to support the assessment of NAFLD.

Qualified radiologists performed ultrasonographic evaluation of hepatic steatosis using a standardized grading system. Grade 1 indicated mild echogenicity with normal visualization of hepatic vessels and the diaphragm. Grade 2 showed moderate echogenicity with partial obscuration of these structures. Grade 3 was marked echogenicity with poor visualization of both hepatic vessels and the diaphragm. A second radiologist independently evaluated a random subset of 10 cases to assess inter-rater reliability in ultrasound grading, and Cohen’s kappa coefficient was calculated. A kappa value greater than 0.80 was considered indicative of excellent agreement.

Missing data were handled by excluding participants with incomplete ultrasound or biochemical results from the final analysis. Outliers in continuous variables were identified using the interquartile range (IQR) method. Values exceeding 1.5 times the IQR were verified against source records, and only those confirmed correct were retained for analysis.

Statistical analysis

Descriptive statistics were presented as mean ± SD for continuous variables. Associations between categorical variables were assessed using the chi-squared test. Differences in means across groups were analyzed using analysis of variance (ANOVA) or t-tests as appropriate. A p-value of less than 0.05 was considered statistically significant. Data analysis was performed using IBM SPSS Statistics v22 (IBM Corp., Armonk, USA) and Microsoft Excel (Microsoft Corp., Redmond, USA).

## Results

A total of 26 obese patients were included in the study. The population showed a predominance of participants in the 31-40 year age group. A slight female preponderance was noted, with 14 (53.8%) female participants (Table 1).

**Table 1:** Age and gender distribution

Age group (years)	Male (n=12)	Female (n=14)	Total (n=26)
18-30	2 (16.6%)	3 (21.4%)	5 (19.2%)
31-40	4 (33.3%)	5 (35.7%)	9 (34.6%)
41-50	3 (25%)	4 (28.5%)	7 (26.9%)
>50	3 (25%)	2 (14.2%)	5 (19.2%)

Among the 26 participants, 18 (69.2%) were classified as obese with a BMI ≥30 kg/m². The most commonly observed fatty liver grade was Grade II, found in 11 (42.3%) individuals. Moderate to severe fatty liver (Grades II and III) was observed in 17 (65.4%) participants. High AIP values (>0.21) were present in 16 (61.5%) patients, and 23 (88.4%) had elevated atherogenic risk. AIP levels showed a statistically significant increase with fatty liver severity (p<0.05). Similarly, the mean BMI rose progressively across fatty liver grades (p<0.05) (Table 2).

**Table 2:** BMI distribution BMI: body mass index

BMI category (kg/m²)	No. of patients	Percentage (%)
25-29.9 (Overweight)	8	30.8%
30-34.9 (Obese Class I)	10	38.5%
35-39.9 (Obese Class II)	5	19.2%
≥40 (Obese Class III)	3	11.5%

Regarding fatty liver grading, Grade II was the most common, observed in 11 (42.3%) patients, followed by Grade I in nine (34.6%) patients and Grade III in six (23.1%) patients. In total, 17 (65.4%) patients had moderate to severe fatty liver (Grades II and III), indicating a substantial burden of progressive hepatic steatosis among obese individuals. These findings suggest a trend toward advanced stages of fatty liver disease, even in a relatively small cohort (Table 3).

**Table 3:** Ultrasound grading of fatty liver

Fatty liver grade	No. of patients	Percentage (%)
Grade I	9	34.6%
Grade II	11	42.3%
Grade III	6	23.1%

A majority of the patients, 16 (61.5%), had AIP values greater than 0.21, placing them in the high-risk category for atherosclerosis and cardiovascular disease. Seven (26.9%) patients had intermediate risk (AIP 0.11-0.21), while only three (11.5%) fell into the low-risk category (AIP <0.11). Notably, 23 (88.4%) obese participants demonstrated elevated AIP values, highlighting the strong association between obesity and increased atherogenic potential. These findings support using AIP as a practical biomarker for early cardiovascular risk assessment in MASLD patients with elevated BMI (Table 4).

**Table 4:** AIP distribution AIP: atherogenic index of plasma

AIP range	No. of patients	Percentage (%)	Risk interpretation
<0.11	3	11.5%	Low risk
0.11-0.21	7	26.9%	Intermediate risk
>0.21	16	61.5%	High risk

The mean total serum bilirubin was 0.98 ± 0.21 mg/dL, with two patients (7.7%) showing values above the reference range. Direct bilirubin levels averaged 0.31 ± 0.09 mg/dL, with only one patient (3.8%) exhibiting elevated levels. The mean serum glutamic-oxaloacetic transaminase (SGOT/AST) was 42.5 ± 15.3 IU/L, exceeding the upper limit of normal in nine patients (34.6%). Serum glutamic-pyruvic transaminase (SGPT/ALT) had a mean value of 58.2 ± 20.7 IU/L, with elevated levels observed in 11 patients (42.3%). Alkaline phosphatase levels were within normal limits for most participants, with a mean of 116.3 ± 28.5 IU/L, and abnormal values were found in three patients (11.5%). The mean serum albumin and globulin levels were 3.9 ± 0.5 g/dL and 2.8 ± 0.4 g/dL, respectively, with hypoalbuminemia observed in two patients (7.7%) and no abnormalities in globulin levels. The albumin-to-globulin (A/G) ratio was within the normal range in all participants, with a mean value of 1.4 ± 0.3 (Table 5).

**Table 5:** LFT parameters among study participants (n=26) SGOT (AST): serum glutamic-oxaloacetic transaminase (aspartate aminotransferase); SGPT (ALT): serum glutamic-pyruvic transaminase (alanine aminotransferase); A/G ratio: albumin to globulin ratio (compares the amount of albumin to globulin in the blood); LFT: liver function test

Parameter	Mean ± SD	Reference range	No. of patients with abnormal values
Serum bilirubin (Total)	0.98 ± 0.21 mg/dL	0.3-1.2 mg/dL	2 (7.7%)
Serum bilirubin (Direct)	0.31 ± 0.09 mg/dL	0.1-0.4 mg/dL	1 (3.8%)
SGOT (AST)	42.5 ± 15.3 IU/L	10-40 IU/L	9 (34.6%)
SGPT (ALT)	58.2 ± 20.7 IU/L	7-56 IU/L	11 (42.3%)
Alkaline phosphatase	116.3 ± 28.5 IU/L	40-129 IU/L	3 (11.5%)
Serum albumin	3.9 ± 0.5 g/dL	3.5-5.0 g/dL	2 (7.7%)
Serum globulin	2.8 ± 0.4 g/dL	2.3-3.5 g/dL	0 (0%)
A/G ratio	1.4 ± 0.3	1.0-2.5	0 (0%)

Statistical analysis revealed significant differences (p<0.05) in mean AIP values across the three grades of fatty liver. This progressive rise in AIP with increasing hepatic steatosis severity supports its potential as a non-invasive indicator for tracking MASLD progression (Table 6).

**Table 6:** Correlation of AIP with grades of fatty liver AIP: atherogenic index of plasma; ANOVA: anlaysis of variance

Fatty liver grade	Mean AIP ± SD	ANOVA F-value	ANOVA p-value
Grade I	0.19 ± 0.04	17.54	0.00002
Grade II	0.27 ± 0.05		
Grade III	0.35 ± 0.07		

A similar trend was observed in BMI, with mean values increasing across fatty liver grades: Grade I, 29.2 ± 2.3 kg/m²; Grade II, 32.1 ± 3.1 kg/m²; and Grade III, 35.6 ± 2.8 kg/m². ANOVA testing confirmed the statistical significance of these differences (p<0.05), demonstrating a strong correlation between increasing BMI and worsening hepatic steatosis. These results reinforce the importance of monitoring BMI in obese individuals to prevent the progression of MASLD (Table 7).

**Table 7:** Correlation of BMI with grade of fatty liver BMI: body mass index; ANOVA: anlaysis of variance

Fatty liver grade	Mean BMI ± SD (kg/m²)	ANOVA F-value	ANOVA p-value
Grade I	29.2 ± 2.3	11.12	0.0004
Grade II	32.1 ± 3.1		
Grade III	35.6 ± 2.8		

## Discussion

This study investigated the relationship between AIP, BMI, and ultrasound-diagnosed fatty liver disease in obese patients, yielding critical clinical insights. The data demonstrate that AIP and BMI may serve as valuable indicators for assessing hepatic steatosis severity in this population.

AIP, calculated as the logarithm of the TG to HDL-C ratio, reflects atherogenic and protective lipid fractions and serves as a dynamic indicator of metabolic health. Dobiásová and Frohlich [10], who introduced AIP, demonstrated its strong association with small, dense low-density lipoprotein (LDL) particles and cardiovascular risk mechanisms overlapping with MASLD.

Grade II fatty liver was the most prevalent (42.3%) among 26 obese participants, followed by Grade I (34.6%) and Grade III (23.1%). These findings suggest a trend toward moderate hepatic steatosis, consistent with the results of Xing et al. [1], who reported a statistically significant (p<0.05) positive correlation between increasing BMI and hepatic fat accumulation.

BMI distribution showed that most participants fell into the Obese Class I and II categories, with mean BMI values of 32.1 kg/m² in Grade II and 35.6 kg/m² in Grade III fatty liver. The progressive increase in BMI with fatty liver severity was statistically significant (p<0.05), aligning with previous studies by Polyzos et al. [2] and Bays et al. [3], who described the pathogenic role of adipocyte hypertrophy and visceral fat in NAFLD development.

Regarding AIP, the majority of participants (61.5%) were classified as high risk (AIP >0.21), while 26.9% fell into the intermediate-risk group and 11.5% into the low-risk group. Mean AIP values increased progressively with fatty liver severity: 0.19 in Grade I, 0.27 in Grade II, and 0.35 in Grade III (p<0.05). These findings are consistent with research by Wang et al. [4], who identified AIP as a strong predictor of MASLD in obese individuals. Zhu et al. [6] reported that AIP outperformed traditional lipid parameters in predicting liver dysfunction in a large Chinese cohort. These results support using AIP as a simple, cost-effective biomarker for assessing MASLD progression, particularly in resource-limited settings.

This study further supports the role of AIP in predicting both hepatic and cardiovascular risk. Akbas et al. [7] found AIP to correlate with cardiovascular risk in diabetic populations, emphasizing its broader metabolic relevance. Given that MASLD is closely linked with increased cardiovascular morbidity, AIP’s ability to assess both hepatic and vascular risk makes it particularly clinically significant.

Although BMI and AIP independently correlate with the severity of fatty liver, their combined use may offer a more comprehensive predictive model. Kotronen et al. [8] proposed that hepatic fat accumulation results from total body fat and lipotoxicity caused by elevated circulating free fatty acids, which are indirectly reflected by AIP levels. Dong et al. [9] reported a positive association between AIP and MASLD among Chinese and Japanese populations, suggesting that AIP may serve as a novel screening indicator for non-obese individuals with MASLD across different countries. Fadaei et al. [11] found that AIP levels were significantly higher in NAFLD patients than in healthy controls and that AIP independently correlated with carotid intima-media thickness in MASLD patients. Our findings are consistent with those of Ünal et al. [12], who reported a strong association between AIP and hepatic steatosis. They emphasized that AIP is a simple, cost-effective, and easily calculable metric that can be a practical tool for predicting MASLD and cardiovascular disease risk in obese patients.

Emerging evidence also links AIP with broader metabolic disturbances, such as insulin resistance and chronic inflammation, contributing to NAFLD pathogenesis. Zheng et al. [13] reported significant correlations between AIP, homeostatic model assessment of insulin resistance (HOMA-IR), and inflammatory markers such as C-reactive protein, further supporting its role as a comprehensive metabolic risk marker.

While newer non-invasive tools like the MASLD Liver Fat Score (LFS) (previously known as NAFLD-LFS) and Fibrosis-4 (FIB-4) index have been developed, they often require multiple biochemical parameters. In contrast, AIP and BMI are simple, cost-effective, and practical for use in primary care, as supported by Liu and Lu [14] in a Korean population study. This is particularly important given the progressive nature of MASLD, which can advance to cirrhosis, as highlighted by Younossi et al. [15], who called for improved early detection strategies.

Demographic variables may influence these associations. Cai et al. [16] reported sex-specific differences, with stronger AIP-MASLD correlations in male patients, potentially due to hormonal influences and variations in fat distribution. Further research is warranted to explore these factors for more personalized risk stratification.

Lifestyle modification remains key from a preventive perspective. Weight loss and lipid control can reverse early MASLD and reduce AIP values. Vilar-Gomez et al. [17] demonstrated histological improvement in MASH patients who achieved weight loss of over 7% through lifestyle interventions.

Limitations

This study has several limitations. The small sample size (n=26) limits generalizability, and its single-center design may not reflect population diversity. The cross-sectional design precludes conclusions about causality. Although practical, ultrasound has lower diagnostic accuracy than advanced imaging or biopsy. The absence of multivariate analysis restricts the ability to adjust for potential confounders and may lead to oversimplification of observed associations.

Additionally, liver enzymes and other biochemical markers were not evaluated, limiting the assessment of liver function. This omission is significant in NAFLD, where transaminase levels (ALT and AST) and markers like GGT provide critical insights into hepatic inflammation and injury. Lifestyle factors such as diet, physical activity, alcohol intake, and medication use were not accounted for despite their influence on lipid metabolism and hepatic fat accumulation. Potential confounding variables such as genetic predisposition or other metabolic conditions were also not controlled for, which may have influenced the observed associations. Furthermore, the single-timepoint measurement of biochemical parameters and anthropometrics may not capture temporal variations, limiting the robustness of the data.

## Conclusions

This investigation identifies BMI and AIP as potentially useful markers for assessing the severity of fatty liver in obese individuals. The significant association observed between these parameters and ultrasonographic grading of hepatic steatosis suggests a role for their further exploration in MASLD risk stratification. However, given the small sample size, single-center setting, and cross-sectional design, these findings should be interpreted cautiously. The utility of BMI and AIP in routine screening protocols remains a promising avenue, particularly in resource-limited settings, but should not be adopted without further validation. Future longitudinal and multicenter studies with larger cohorts and multivariate analysis are necessary to confirm these associations, determine their predictive value, and establish causality.
